# Does health intervention research have real world policy and practice impacts: testing a new impact assessment tool

**DOI:** 10.1186/1478-4505-13-3

**Published:** 2015-01-01

**Authors:** Gillian Cohen, Jacqueline Schroeder, Robyn Newson, Lesley King, Lucie Rychetnik, Andrew J Milat, Adrian E Bauman, Sally Redman, Simon Chapman

**Affiliations:** School of Public Health, University of Sydney, Edward Ford Building (A27), Fisher Rd, Sydney, NSW 2006 Australia; Prevention Research Collaboration, School of Public Health, University of Sydney, level 6, Building D17, Sydney, NSW 2006 Australia; School of Medicine, University of Notre Dame, 160 Oxford Street, Darlinghurst Sydney, NSW 2010 Australia; Centre for Epidemiology and Evidence, NSW Ministry of Health, Level 7, 73 Miller St, North Sydney, NSW 2060 Australia; Sax Institute, Level 13, Building 10, 235 Jones Street, Ultimo, NSW 2007 Australia

**Keywords:** Intervention research, Policy, Research impact, Research translation

## Abstract

**Background:**

There is a growing emphasis on the importance of research having demonstrable public benefit. Measurements of the impacts of research are therefore needed. We applied a modified impact assessment process that builds on best practice to 5 years (2003–2007) of intervention research funded by Australia’s National Health and Medical Research Council to determine if these studies had post-research real-world policy and practice impacts.

**Methods:**

We used a mixed method sequential methodology whereby chief investigators of eligible intervention studies who completed two surveys and an interview were included in our final sample (n = 50), on which we conducted post-research impact assessments. Data from the surveys and interviews were triangulated with additional information obtained from documentary analysis to develop comprehensive case studies. These case studies were then summarized and the reported impacts were scored by an expert panel using criteria for four impact dimensions: corroboration; attribution, reach, and importance.

**Results:**

Nineteen (38%) of the cases in our final sample were found to have had policy and practice impacts, with an even distribution of high, medium, and low impact scores. While the tool facilitated a rigorous and explicit criterion-based assessment of post-research impacts, it was not always possible to obtain evidence using documentary analysis to corroborate the impacts reported in chief investigator interviews.

**Conclusions:**

While policy and practice is ideally informed by reviews of evidence, some intervention research can and does have real world impacts that can be attributed to single studies. We recommend impact assessments apply explicit criteria to consider the corroboration, attribution, reach, and importance of reported impacts on policy and practice. Impact assessments should also allow sufficient time between impact data collection and completion of the original research and include mechanisms to obtain end-user input to corroborate claims and reduce biases that result from seeking information from researchers only.

## Background

Since the 1980s there has been a growing expectation that health research will have direct social and economic utility and impact [1–5]. Health intervention research, in particular, which uses scientific methods to produce knowledge about treatments, services, programs, or strategies that aim to protect, promote, or improve health, is assumed to hold immediate promise for influencing and improving future policy and practice [2, 6]. Research funding submissions require applicants to predict the practical benefits that might flow from their planned studies [7, 8]. However, a large proportion of funded research fails to translate into real world solutions [6, 9–12].

Over 90 different terms are used to describe research impact on policy and practice [13], including translation, diffusion, adoption, adaptation, uptake, exchange, research utilization, and research implementation. How and to what extent research is translated into policy and practice is emerging as an important field of research [14, 15]. A key area within this field is assessment of impact, or how to measure the dividends from research [14, 16].

In practice, assessments of research impacts are mostly commissioned by governments to determine the public benefit from research spending [4, 15, 17]. Governments are increasingly signaling that research metrics of research quality are insufficient to determine research value because they say little about real-world benefits of research [3, 4, 7].

To date, gold standards in the assessment of research impact combine case study narratives with relevant qualitative and quantitative indicators [4, 16–18]. The impact assessment literature universally calls for expanded measures to better assess the nature and quality of real world impacts, as well as better predictive measures of longer term benefits [4, 14]. “*The holy grail is to find short term indicators that can be measured before, during or immediately after the research is completed and that are robust predictors of the longer term impact … from the research*” [14].

In this paper, we build on the existing literature to propose an expanded ‘impact assessment’ framework, and apply a model which builds on current best practice to 5 years (2003–2007) of intervention research funded by Australia’s peak health and medical research funding agency, the National Health and Medical Research Council (NHMRC) [15, 18–24].

Our primary aims were to i) pilot a modified impact assessment process and ii) determine what proportion of intervention projects had any demonstrable impact on subsequent policy or practice in ‘real world’ settings after the research undertaken had concluded and to group these projects according to the magnitude their impacts. The project was approved by the University of Sydney Human Research Ethics Committee (15003).

## Methods

### Conceptual framework

A number of approaches have been developed to describe the impacts of research [15]. The Payback framework [19, 24] and its adaptation into the Canadian framework [20] are the most widely used [15]. These approaches and the literature underpinning them indicate that it is important for impact assessment to distinguish between different types and stages of impact and to draw on multiple sources of data. A conceptual framework can help organize data collection, analysis, and reporting to promote clarity and consistency in the impact assessments made. It is important to acknowledge that such a model facilitates impact assessment rather than being a precise model of how research impact occurs [25].

**Figure 1:**
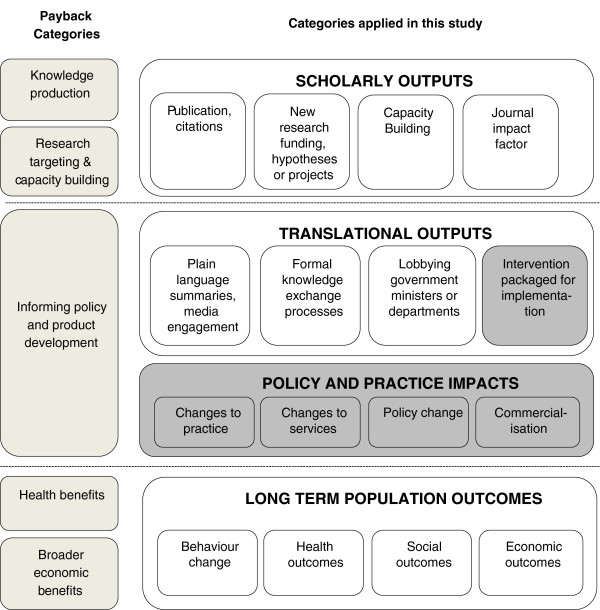
**Comparing categories of research impacts across models.** *The dark grey shaded areas represent the impacts of relevance to this study.

The primary focus of our study was to examine the policy and practice impacts of research that occur in the ‘real world’ after the research had concluded. We combined and adapted the Payback and Canadian frameworks to produce a conceptual model that would best fit our purpose. We grouped different types of impacts into four levels of impact that might arise from intervention research: i) scholarly outputs; ii) translational outputs; iii) policy or practice impacts; and iv) long-term population outcomes. Each impact level was populated with sub-categories or indicators to further facilitate assessment of the type of impacts that occur at each level (Figure 1). For comparison with our approach, the Payback categories are included in Figure 1 alongside the impact levels we describe.

We considered that the scholarly outputs, such as publications, acquisition of new research funding, and research capacity building, were not real-world impacts as we had defined them and therefore they are not reported here; others have made a similar distinction [21].

Translational outputs were defined as those activities that occur beyond research publication and are designed to facilitate uptake of study findings in real-world settings. These outputs may be activities undertaken by researchers, their institutions, or government programs to facilitate knowledge uptake such as implementation protocols, training workshops, and information exchange meetings. They may also be part of a general dissemination strategy conducted at the end of a study. These outputs may or may not lead to policy or practice impacts and long-term population changes. While some impacts at this level, particularly those related to information packaging for implementation, are included as part of our impact assessment here, we considered these to be of lesser importance than policy or practice impacts.

Policy or practice impacts were defined as demonstrable changes, or benefits to products, processes, policies, and or practices, that occur after a research project has concluded. These impacts are concrete, measurable changes in policy or practice such as a new government policy, a change in organizational or clinical practice, a health education campaign or related new funding that can be attributed to the research intervention in question. Impacts at this level could also include stopping or changing existing interventions following demonstration of intervention ineffectiveness. Policy or practice impacts can be widespread or localized, and may benefit specific or general populations. Impacts at this level are our primary focus in this paper.

Finally, we defined long-term population outcomes as changes in health behaviors and health outcomes, such as disease incidence, prevalence, or other health indicators, or as improvements in social or economic outcomes. Such changes rarely occur quickly, and they may be difficult to attribute to health intervention research, let alone to single studies [9, 15]. Due to these attribution issues and the distal nature of population outcomes, outcomes at this level were not assessed in our research.

As in other frameworks [20, 24], our model (Figure 1) reflects a sequence of impacts (while recognizing that this will often not occur) and distinguishes immediate research outputs from impacts which tend to occur later and beyond the research setting or context. Models with sequential stages assume that an impact or output at one stage may, or may not, lead to increasingly concrete and widespread impacts over time.

### Sampling

Our sample of intervention research studies was generated from a list provided by the NHMRC of 721 primary research grants that were potentially intervention-related (as identified by the NHMRC from their records) which commenced between 2003 and 2007. Studies were included in our sample if they met our definition of intervention research (i.e., any form of trial or evaluation of a service, program, or strategy aimed at disease, injury, or mental health prevention, health promotion or psychotherapeutic intervention, conducted with general or special populations, or in clinical or institutional settings), if the study commenced before 2007, and if the study data had been collected and analyzed before we began our data collection in 2012. Clinical trials of potentially prescribable drugs, vaccines, and diagnostic tests were excluded because of the very different trajectories that such therapeutic goods are required to navigate before being authorized for use by the Australian Therapeutic Goods Administration. We determined if studies met our inclusion criteria initially by reviewing grant documents and publications, after which 83 studies were included in our sample, and then by surveying the chief investigators (CIs), after which 13 studies from our initial sample were found to be ineligible, leaving 70 eligible studies. We chose to include intervention studies commencing between 2003 and 2007 in our sample because this 5-year window provided a balance between allowing projects sufficient time post-study completion to have realized real-world impacts by the time of our data collection in 2012 and to minimize recall bias about grants that finished too long ago.

**Figure 2:**
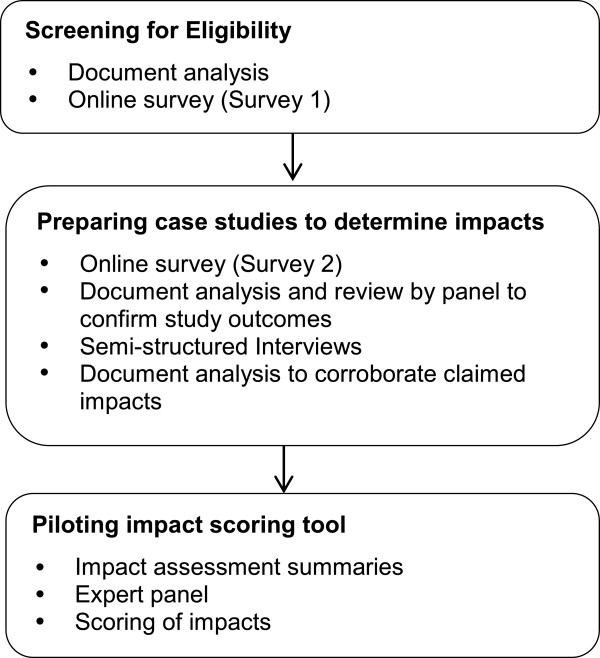
**Overview of study methods.**

Our study used a mixed methods sequential methodology. CIs were invited to complete two surveys and an interview (Figure 2). Only those CIs who completed both surveys and the interview were included in our final sample (n = 50).

### Preparing case studies to determine impacts

In order to determine if the studies had included real-world impacts relevant to our study we collected data from multiple data sources (Figure 2 and described below) which were triangulated to produce case studies. The case studies were reviewed by two authors and either classified as having at least one impact or no impacts.

### Data sources

#### Online survey

An online survey (see http://hdl.handle.net/2123/9864) was sent to study CIs to elicit their views about the potential influence and significance of their research findings and any impacts of their research in the real world following the research project. They were also asked questions on contextual factors and research characteristics known to be influential in practice uptake.

#### Independent confirmation of results

Information about the research findings of each study was considered to be essential in determining the extent to which the reported post-study impacts could be attributed to the specific research projects under consideration. Rather than only relying on outcomes reported by the CIs, which may have been subject to responders’ bias and selective recall [26], the information related to the study findings included in the case studies was subject to a separate verification process. This involved developing a summary for each study based on published results related to the principal study outcomes which was then reviewed by a panel of authors. The panel classified interventions according to whether there were statistically significant changes on principal outcomes: those that did, those with ‘mixed’ results (in cases where there were significant changes for some but not all principal outcomes or if unintended or ancillary results were emphasized over the primary focus of the original research questions), and those where the intervention did not produce statistically significant changes. In some cases, no published results related to the principal outcomes could be found. The panel was not asked to make judgments in relation to the quality of the research, the appropriateness of the research methods, or the importance of the findings. In this assessment and verification step, ‘statistical significance’ provided a simple and objective way of considering study outcomes, and was not intended as a measure of the clinical or societal value of the outcome.

#### Semi-structured interviews

Semi-structured ‘conversational’ interviews were customized for each study based on responses to the online survey. The interviewers sought to obtain more information about any claimed real-world impacts of the study and to explore the CIs perceptions of what had helped or hindered the uptake of their intervention. Interviews covered the following themes: i) implications: what are they and have any come into effect?; ii) impacts: what occurred, and why do you think some impacts occurred, while others did not?; iii) engagement with potential end-users (research team and other stakeholders) before, during, and after the study; iv) communication before, during, and after the completion of the study; v) contribution to knowledge: what was the nature of any contribution?; and vi) follow-up: what occurred following the post-research dissemination?

#### Document analysis to corroborate reported impacts

The impacts reported by the CIs were corroborated, where possible, by completing an internet search using Google to search for relevant web pages, newsletters, media releases, or other documents.

### Piloting the impact scoring tool

We sought to pilot an impact scoring process so that we could group and compare studies according to the magnitude of their impact. This process included the following steps:

#### Preparing impact assessment summaries

The detailed case studies that included real-world impacts relevant to our study (n = 19) were used to develop a two to three page impact assessment summary for consideration by an expert panel. A common format was used to allow comparison between cases. The summaries included the study aims and research question/s, a description of the intervention, the study findings, post-study impacts that potentially met our definition of policy and practice impacts (only impacts that had actually occurred rather than those that could potentially occur in the future were included), evidence of independent corroboration and attribution of impacts, any contextual factors that may have had an influence on impact, and a summary of information and evidence related to each of our scoring criteria (corroboration, attribution, reach, and importance) for each of the reported impacts (see scoring of impacts below).

#### Convening an expert panel

An expert panel made up of 12 experienced intervention researchers from the fields of public health, health policy, and clinical medicine, 6 of whom were external to the project, was convened to review and assess the impact assessment summaries and provide an overall assessment of the policy and practice impact of each of study. Four panelists were former or current government health policy makers. As the studies being assessed were heterogeneous in terms of the topic area they addressed, it was not possible to convene a panel made up of content experts. Panelists were therefore selected because of their intervention research expertise and their knowledge of how evidence is translated into policy and practice. None of the panelists had been involved in the studies being assessed.

To introduce the session, the panel was briefed on our study methods. They were also introduced to our conceptual framework and provided with our definition of policy and practice impacts. Each case study was then presented in turn. After each presentation, panelists were given an opportunity to ask clarifying questions before scoring impact for that case. Panel members were provided with the case summaries in advance and hard copies of these were made available to the panelists for reference during the panel proceedings. Panel proceedings were completed over the course of a single day.

#### Scoring of impacts

The conceptual framework (Figure 1) was used to focus the panel’s attention on the impacts of interest for our study during the scoring process. Only impacts that were consistent with the categories of interest within this model were scored. The combined impacts of each case were scored using a modification of the scoring tool used by the Australian Excellence in Innovation (EII) trial [22] and the UK’s Research Excellence Framework trials [27, 28]. The original processes considered intervention reach combined with significance of impact; however, a recommendation from the EII trial was that reach and impact significance should be judged separately to avoid biasing small studies [22]. The need for also considering corroboration and attribution of claimed impacts has been repeatedly emphasized in the literature [4, 14, 16, 17, 22].

Based on these recommendations we developed a scoring system that included four dimensions of impact, namely corroboration, attribution, reach, and importance. Importance was used as it was felt the term significance may be confused with the statistical significance of the study findings rather than the importance of the reported impacts. Our scoring system consisted of a series of questions considered by the panelists when scoring each dimension of impact and a rating system based on a Likert scale of 1 to 9 for scoring each dimension (Table 1). We also provided panelists with specific instructions in relation to scoring each dimension. For example, we instructed the panel to judge reach of the impacts against what was possible for the relevant target population, not against other interventions with different potential, and to judge importance of the impacts claimed by referring to the definition and examples in the scale. Using this information as a guide, panelists were asked to score the overall impact of each study, independently, using hand held keypad transmitters.

**Table 1:** **Scoring system**

Independent corroboration	Attribution	Reach	Importance
Did the materials provided to verify the research impact convince the Panel that the key impact claims had been corroborated?	Was the link between the research and the claimed post-research impact clearly demonstrated?	How broad was the reach of the impacts on the relevant constituencies, when reach is defined as spread and breadth of influence post-study?	How important are the post-research impacts to products, processes, behaviors, policies, and/or practices, when importance is defined the significance and noteworthiness of an impact and its ability to endure?
8–9 – Corroborated	8–9 – Significant contribution	8–9 – Extensive reach because it has widespread reach in relevant constituencies in multiple countries	8–9 – Extremely important
6–7 – Probably corroborated	6–7 – Good contribution	6–7 – Broad reach because it has widespread reach in relevant constituencies across multiple regions, or states, in Australia or internationally	6–7 – Very important
5 – Possibly or partially corroborated	5 – Moderate contribution	5 – Moderate reach because it is reaching relevant constituencies in multiple discrete locations	5 – Moderately important
3–4 – Not corroborated but further information could provide a more convincing corroboration	3–4 – Small or some contribution	3–4 – Some reach (modest) because the impact has only modest reach in local constituencies, or has continued in the areas where the study was conducted	3-4 – Some import
1–2 – Not corroborated and it is unlikely that further information could provide a more convincing corroboration	1–2 – There is no discernible link between the underpinning research and the claimed post-study research	1–2 – Limited or no assessable post-study reach	1-2 – Limited or no assessable post-study importance

### Data analysis

The results from the panel were analyzed by examining the distribution of scores as group means and standard deviations for each question, as well as a measure of spread of responses (coefficient of variation, standard deviation/mean). The distribution was also categorized into tertiles: low, medium, and high impacts.

## Results

### Response rate

A total of 50 CIs completed both surveys and an interview, providing a response rate of 71% (50 out of a possible 70 eligible studies). Of the 20 CIs that did not respond, 3 did not return Survey 1 (83 sent; 7 confirmed as ineligible), 11 did not respond after receiving Survey 2 (73 sent; 6 confirmed as ineligible), and 6 did not respond to a request for interview (56 invitations sent).

### Number of studies with impacts and other study characteristics

Thirty-one studies (62%) were classified as having no real-world impacts and 19 (38%) were assessed as having at least one impact.

The 50 studies included in our overall sample represent a diverse range of intervention research projects. Just over a third (36%) were primary prevention or health promotion interventions; 24% were early intervention or screening interventions; and 40% were interventions related to the treatment or management of an illness, disease, or disorder. Table 2 provides an overview of the topic areas addressed by the studies within each of these intervention groups for impact and no impact categories. The proportion of primary prevention or health promotion interventions in the no impact group (n = 15; 48%) was greater than that for the impact group (n = 3; 16%).

**Table 2:** **Type of interventions included in our sample by impact category (n = 50)**

Impact category	Primary prevention/Health promotion	Early intervention/Screening	Treatment/management of an illness/disease/disorder
	n = 18 (36%)	n = 12 (24%)	n = 20 (40%)
No impact	n = 15 (48%)*	n = 5 (16%)	n = 11 (36%)
n = 31 (62%)	Adolescent Mental Health	Childhood obesity	Alcohol misuse
	Alcohol	Family violence	Allergy/Asthma
	Allergy prevention	Parenting skills	Anorexia
	Childhood injury (ATSI)	Premature infants	Arthritis (2)
	Childhood obesity (Low SES)	Suicide SUD	Asthma
	Falls prevention (3)		Cancer (2)
	Healthy ageing (2)		Diabetes
	Hospital acquired infection		Post-traumatic stress
	Sports injuries		Renal disease
	Tobacco (ATSI) (2)		
	Tobacco (CALD)		
Impact	n = 3 (16%)*	n = 7 (37%)	n = 9 (47%)
n = 19 (38%)	Adolescent health	Bowel cancer (2)	Anorexia
	Falls prevention (2)	Chronic disease (ATSI)	Arthritis (2)
		Language delay	Childhood obesity
		Maternal & infant health	Dental decay
		Parenting skills (2)	Depression
			Neck Pain
			Obsessive compulsive disorder
			Post-traumatic stress

*Fishers exact test *P* = 0.03.

ATSI, Aboriginal and Torres Strait Islander; CALD, Culturally and Linguistically Diverse; SES, Socio-economic status; SUD: Substance Use Disorder.

The funding period for the studies in our final sample ranged from 1 to 4 years, with the mean funding period being 2 years. A large proportion (n = 20; 40%) of studies in our sample did not commence until 2007, the final year in our study period (2003–2007). For most of the studies (88%; n = 44), the grant funding period concluded by 2009, while the funding period for all of the studies (n = 50) had concluded by 2011. The pattern in terms of number of studies by year in which the funding was completed was similar for the impact and no impact categories.

### Results of the impact scoring tool pilot

#### Spread of impact scores

**Figure 3:**
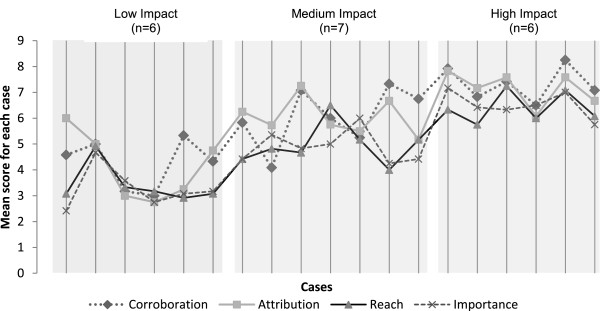
**Mean impact scores for projects with impact for each impact dimension (n = 19).**

For the studies assessed as having impacts (n = 19), the mean impact scores (n = 12 raters) and 95% confidence intervals for each of the impact dimensions within our scoring system (corroboration, attribution, reach, and importance) are provided in Table 3. Responses for each dimension were ranked and divided into tertiles and summarized across dimension in the far right column, as low (n = 6), medium (n = 7), and high (n = 6) impact groups. The four dimensions were not summed to provide a total score for each study as we considered that each dimension should remain as a separate consideration. However, there was general concordance in the ranking of each study across dimensions. The mean scores across cases showed substantial variation, which allowed scores to be categorized into tertiles and these were examined for differences in magnitude of impact between studies Figure 3.

**Table 3:** **Mean impact scores for projects with impact (n = 19)**

Corroboration		Attribution		Reach		Importance		Impact score groups
Mean*	95% CI	Mean*	95% CI	Mean*	95% CI	Mean*	95% CI	
4.6	(3.5–5.7)	6.0	(5.1–6.9)	3.1	(2.3–3.9)	2.4	(1.7–3.2)	Low
5.0	(4.2–5.8)	5.0	(4.3–5.7)	4.9	(4.4–5.4)	4.7	(4.0–5.3)	Low
3.2	(2.8–5.5)	3.0	(2.5–3.5)	3.3	(2.7 – 4.0)	3.6	(2.6–4.5)	Low
3.0	(2.3–3.7)	2.8	(1.9–3.5)	3.2	(2.5–3.8)	2.8	(1.9–3.6)	Low
5.3	(4.0–6.6)	3.3	(2.6–3.9)	2.9	(1.9–3.9)	3.1	(1.8–4.3)	Low
4.3	(3.1–5.6)	4.8	(3.5–6.0)	3.1	(2.3–3.9)	3.2	(2.5–3.9)	Low
5.8	(4.8–6.8)	6.3	(5.6–6.9)	4.4	(3.9–4.9)	4.4	(3.7–5.2)	Medium
4.1	(3.5–4.7)	5.7	(4.7–6.8)	4.8	(4.2–5.4)	5.4	(4.3–6.4)	Medium
7.1	(6.5–7.7)	7.3	(6.5–8.0)	4.7	(3.9–5.5)	4.8	(3.7–6.0)	Medium
6.0	(5.3–6.7)	5.8	(5.0–6.5)	6.5	(5.6–7.4)	5.0	(4.2–5.8)	Medium
5.3	(4.4–6.1)	5.5	(4.5–6.5)	5.2	(4.4–5.9)	6.0	(5.2–6.8)	Medium
7.3	(6.5–8.2)	6.7	(6.7–7.7)	4.0	(3.53-4.5)	4.3	(3.6–4.9)	Medium
6.6	(5.7–7.8)	5.2	(4.3–6.0)	5.2	(4.4–5.92)	4.4	(4.0–4.8)	Medium
7.9	(7.3–8.5)	7.8	(7.9–8.3)	6.3	(5.9–6.6)	7.2	(6.5–7.8)	High
6.8	(6.2–7.4)	7.2	(6.5–7.8)	5.8	(4.9–6.6)	6.4	(5.7–7.2)	High
7.4	(6.6–8.2)	7.6	(6.9–8.3)	7.3	(6.6–7.9)	6.3	(5.5–7.2)	High
6.5	(5.8–7.2)	6.1	(5.3–6.9)	6.0	(5.1–6.9)	6.5	(5.7–7.3)	High
8.3	(7.8–8.7)	7.6	(7.2–8.0)	7.1	(6.5–7.7)	7.0	(6.3–7.6)	High
7.1	(6.5–7.6)	6.7	(5.9–7.4)	6.1	(5.5–6.7)	5.8	(4.9–6.6)	High

*12 raters. CI, Confidence interval.

#### Distribution of responses among raters

The coefficient of variation between raters’ scores for each grant within each impact dimension was generally small, indicating that there was a high degree of consistency of scores between raters. The variation for ratings was less than 0.25 for all of the projects in the high (n = 6) and medium level (n = 7) impact groups. However, it ranged from 0.39 to 0.41 in the low impact group (n = 6), indicating that there was less agreement between raters for these studies, which tended to have less corroborative evidence about impact, or were assessed as being closer to traditional research metric impacts than to policy and practice impacts. This showed greater spread in the distribution of panel responses in a subset of grants appraised.

### Type of impacts reported

The type of impacts reported for the 19 studies classified as having impacts are summarized by impact score group in Table 4. Some studies reported impacts of more than one impact type (e.g., policy and service change). Half of the impacts (n = 21) reported were in the form of translational outputs (e.g., intervention resources, including websites, publications, and manuals for end users or training). Although potentially useful resources for policy and practice, resources were not necessarily proof of use and we considered them to be the lowest level of impact. Where these impacts were endorsed by professional bodies or had significant reach, the impacts were considered to be of higher magnitude. The remaining impacts (n = 21) were classified as policy and practice impacts which included clinical practice changes (n = 6), service changes (n = 9), organizational changes (n = 1), commercialization of products or services (n = 1), and policy changes (n = 4). As these all require a more substantial degree of personal or organizational change, they were considered to equate to a higher level of impact.

**Table 4:** **Type of impacts by impact score group (n = 19)**

Impact category		Number impacts by impact category and impact score			Example	Total number impacts
		High impact studies,*	Medium impact studies,*	Low impact studies,*		
		n = 6	n = 7	n = 6		
Policy and practice impacts	Policy changes	3	0	1	A school-based parent education program to promote adolescent health influenced the 2011 change to the Victorian Liquor Control Reform Act 1998 (secondary supply)	4
	Organizational change	1	0	0	An intervention targeting the year before and after birth in Aboriginal children in remote areas led to improvements in continuity of care between the hospital system and remote community care	1
	Commercial product or service	1	0	0	The license for a parenting program that was shown to be effective for Indigenous Families was sold to a province in Canada where it is still in operation and has been formally evaluated	1
	Service changes	4	3	2	An intervention to provide more effective communication to improve participation in bowel cancer screening led to an advanced notification letter being included in the National Bowel Cancer Screening Program in Australia. An advanced notification letter has been adopted by at least four other countries around the world	9
	Clinical practice changes	1	4	1	An intervention to retain the neck muscles of neck pain patients had led to changes to clinical practice	6
Total number policy & practice impacts reported						21*
Translational outputs	Professional training (e.g., College of Practitioners)	2	5	2	A professional development training program based on an intervention to slow progression of knee osteoarthritis was developed and delivered through peak practitioner bodies	9
	Professionally endorsed documentation (guidelines, manuals)	3	3	1	The findings from a school-based parent education program to promote adolescent health have been included in 2009 Australian guidelines to reduce health risks from drinking alcohol	7
	Intervention resources (websites, lay publications, training manuals)	2	1	2	A protocol for the non-invasive management of tooth decay in private practice was endorsed for implementation at the international level by leaders in the field	5
Total number translational outputs reported						21*

*Each study may have had impacts within more than one impact category (e.g., policy change and clinical practice change, as well as professionally endorsed documentation).

Within the group of studies (n = 6) assessed by the panel to have high impacts, there was one commercial product launch, three policy changes, four changes to service delivery, one organizational change, and one change to clinical practice. All of the studies in the high and medium impact group had at least one impact that fell within the policy and practice impact category, while only four out of six of the studies in the low impact group had impacts of this type. None of the studies in the low and medium impact groups had more than one impact within the policy and practice category, two studies within the high impact group had three impacts each of this type, three had two impacts each of this type, and two had only one impact of this type. Many of the projects classified as having medium (n = 7) and low impact (n = 6) reported impacts that were anecdotal, with weak evidence that was not easily corroborated.

## Discussion

There have been a number of studies that have examined the impacts of a set of publically funded intervention studies, where the unit of measure is an individual study. Our study differs from these in terms of its specific focus on intervention research in clinical or community settings and on measurable policy and practice impacts rather than scholarly outputs or longer term population outcomes [9, 29–31]. We chose to focus on policy and practice impacts as there is a need for reliable measures of impact to provide sound information about translation beyond the research setting, and to counter-balance the tendency to focus on research metrics as a sole indicator of impact. Other studies of health research impacts have defined their scope in terms of the content area (e.g., breast cancer, stroke) [21, 25, 26, 32, 33], or assessed the impacts of a program of research or research institution as a whole [22, 23].

We found that single intervention research studies can and do have concrete and measurable post-research real-world impacts, with 38% of the studies in our sample demonstrating some impacts on policy and practice. The impacts were often multiple and diverse, covering all of the categories of interest within our research impacts model (Figure 1). We also found that the magnitude of impact varied between studies. Studies received lower impact scores where their impacts involved the development of resources and training, rather than concrete changes to policy and practice and/or because evidence to corroborate the researcher’s claims about impact was weak or could not be found. When the studies were divided into tertiles, three almost equal groups (high, medium, and low impact) were formed. In this paper we do not discuss why some studies had impact and others did not. This will be the subject of another paper.

Other research has found that individual studies from a range of research types, including basic, applied, and clinical research, can have wider impacts (outside of research settings) [9, 21, 29]. However, it is difficult to compare our findings with these previous studies due to differences in study methodologies. For example, another Australian study that examined the impacts of intervention research found moderate to high policy and practice impacts were scored in 10 out of 17 cases (59%) [9]. The remaining cases were scored as having limited impact, but the scoring system did not allow a score of zero to be recorded for the impact category. The study also used a broader definition of policy and practice impacts than we did and reported on a more homogeneous sample of interventions. In another study examining the impacts of cardiovascular research (both basic and clinical), all of the 14 clinical cases in the sample had impacts beyond the research setting [33]. In this study, the timeframe from when the original studies were completed to impact data collection was between 15 and 20 years.

The method we piloted resulted in a good level of agreement between raters about the extent of the post-research policy and practice impacts for most of our case studies; there was more variability in scoring of the low impact cases. Our method also provided an estimate of the magnitude of the impact, which is important in order to compare impacts across heterogeneous studies [5]. However, it is possible that not enough time had elapsed for all of the studies in our sample and subsequent impacts may yet occur. It has been suggested that it can take a considerable amount of time (up to 17 years) for evidence to be translated into practice [33, 34]. A considerable proportion (40%) of the studies in our sample commenced in 2007, 5 years before our data collection began in 2012, and nearly half (48%) of the studies were completed in the 3 years prior to our data collection. It is also possible that some of the studies in our sample did not have impacts because they were only single studies. Ideally, policy and practice change should result from a summarized body of knowledge in the form of systematic reviews or research synthesis, rather than single studies alone. It may be that it is more appropriate to apply an impact assessment process to a researcher’s body of work rather than a single research study. This would capture impacts that cannot be attributed to a single research study and allow sufficient time for impacts to occur. We suggest it is important to make explicit the purpose of any research impact assessment process to determine whether the unit of analysis is to be that of a single study, a researcher’s body of work, the research institution, or of a synthesis of all of the published evidence on a given topic.

While others have scored the impacts of research [9, 21, 22, 28, 31], our scoring system is unique in that it involved scoring four separate dimensions of impact, namely corroboration, attribution, reach, and importance. This system sought to overcome some of the issues with attribution and corroboration that had been encountered during previous assessment processes, as well as the need to distinguish reach from significance so as not to downplay the potential impact of smaller studies, or studies with small target groups [4, 14, 16, 17, 22]. However, on reflection, panel members tended to score the importance of the impacts highly across all dimensions, compared to the benchmark examples provided in the scoring sheet. For example, we used the impact of human papilloma virus vaccine research [35, 36] as an example of a study that would be given a high score of nine for importance because of its global implications for prevention of a serious and prevalent disease. None of the research studies in our sample had impacts as significant as this example, but some panel members nevertheless scored some projects at eight for importance. The reasons for this are unclear, although it may be that something of a ceiling effect operates, with raters scoring all projects with impacts above a certain level in terms of importance on a similarly high scale. While the scores may be comparable within any given process, further guidance to panels about appropriate magnitude of scores should be provided to support between-panel comparison. It may also be necessary to review individual panel member scores during the panel process to ensure that all panel members are scoring impacts based on similar considerations [31].

Having a system for scoring cases according to the level of corroborating evidence and degree of evidence for attribution was beneficial. However, it was not possible to find supporting information of acceptable quality for all of the studies in our sample. There was a greater degree of variability in panel scores where limited supporting information was available or it was absent. To improve reliability of scoring, it may be necessary to mark case summaries without adequate supportive evidence as ‘unclassified’ [23] and not score these cases until supporting evidence becomes available. Another way to verify claims made by CIs would be to supplement publicly available information with third party interviews of end-users. This is a resource-intensive process [16] and for this reason few examples where interviews or surveys of end users are used as part of impact assessment processes can be found [9, 37]. To improve the feasibility of this approach, we suggest that end user input should ideally be sought post-panel assessment, so that resources are expended on impacts of potential significance.

Reliance on only the perspectives of researchers is problematic for other reasons. Researchers may sometimes be unaware of post-research impacts of their research, because research impact is seldom a key research performance measure, so they do not actively track uptake, or because they consider that their role finishes with publication [4]. This may result in research impacts being under-reported [26, 29, 30, 32]. Researchers may also differ in the way they conceptualize and explain the impact of their research compared to other groups [38]. In addition, societal importance is a very difficult concept to judge. Therefore, contributors to impact assessment research and impact assessment panels should include as wide a cross section of viewpoints as possible [3, 4, 22]. While our panel included both researchers and practitioners (policy and clinical), limited resources meant that it was still predominantly made up of researchers. The Australian EII trial exemplified end-user participation, with expert panels comprising 70% end users [22]. We recommend that future studies include assessment panels made up of a predominance of non-researchers, and a high mix of different stakeholders.

## Conclusions

There is a growing sense outside, and increasingly inside, the research sector that health intervention research is not an ‘end in itself’, and needs to have demonstrable public benefit. In order to demonstrate this benefit, we need to have a means of measuring the impacts of research. Moreover, if such methods are to be widely used in practice by research funders and academic institutions to assess research impacts, the right balance between comprehensiveness and feasibility must be struck. This study builds on current best practice for assessing real-world policy and practice impacts and demonstrates a systematic and multidimensional approach to impact assessment. The findings of this study could help funding systems determine how to assess impact in the future; however, the methods we employed were resource intensive. Further research to refine the process so that it may be more feasibly applied on a routine basis is warranted.

## Abbreviations

CI: Chief Investigator
EII: Australian Excellence in Innovation (EII) trial
NHMRC: National Health and Medical Research Council.
